# Mechanisms of Neoantigen-Targeted Induction of Pyroptosis and Ferroptosis: From Basic Research to Clinical Applications

**DOI:** 10.3389/fonc.2021.685377

**Published:** 2021-05-27

**Authors:** Jie Yu, Qing Wang, Xiaoyun Zhang, Zhiliang Guo, Xiaodong Cui

**Affiliations:** ^1^ School of Basic Medicine Sciences, Weifang Medical University, Weifang, China; ^2^ The Department of Spine Surgery, The 80th Group Army Hospital of Chinese People’s Liberation Army (PLA) of China, Weifang, China

**Keywords:** neoantigens, pyroptosis, ferroptosis, chimeric antigen receptor T cell (CAR-T), T cell receptor engineered T cell (TCR-T), PD-1/PD-L1, tumor vaccine

## Abstract

Neoantigens are tumor-specific antigens (TSAs) that are only expressed in tumor cells. They are ideal targets enabling T cells to recognize tumor cells and stimulate a potent antitumor immune response. Pyroptosis and ferroptosis are newly discovered types of programmed cell death (PCD) that are different from apoptosis, cell necrosis, and autophagy. Studies of ferroptosis and pyroptosis of cancer cells are increasing, and strategies to modify the tumor microenvironment (TME) through ferroptosis to inhibit the occurrence and development of cancer, improve prognosis, and increase the survival rate are popular research topics. In addition, adoptive T cell therapy (ACT), including chimeric antigen receptor T cell (CAR-T) technology and T cell receptor engineered T cell (TCR-T) technology, and checkpoint blocking tumor immunotherapies (such as anti-PD- 1 and anti-PD-L1 agents), tumor vaccines and other therapeutic technologies that rely on tumor neoantigens are rapidly being developed. In this article, the relationship between neoantigens and pyroptosis and ferroptosis as well as the clinical role of neoantigens is reviewed.

## Introduction

**Figure 1:**
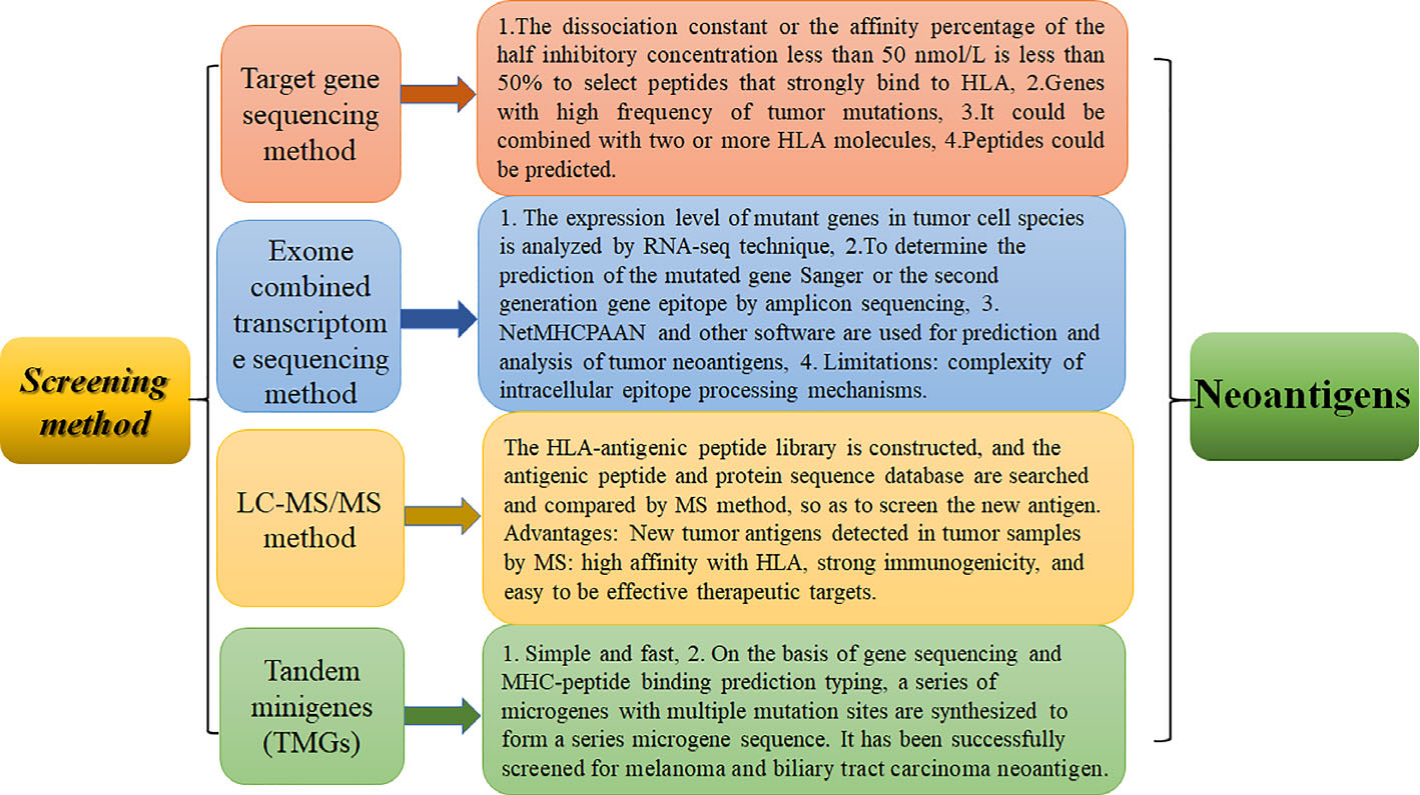
Screening methods of neoantigen identification and their advantages and disadvantages. The advantages and disadvantages of four methods, including serological analysis of recombinant expression cDNA clones, exome combined transcriptome sequencing method, LC-MS/MS method, and tandem microgenre method, are discussed.

Tumors are a major threat to human life and health. Tumor antigens can be divided into tumor-associated antigens (TAAs) and tumor-specific antigens (TSAs). The majority of tumor antigens are TAA-specific embryonic antigens. Different antigens have different levels of immunogenicity, not only low immunogenicity, and it is difficult to induce a long-lasting specific immune response. In addition, TAA-targeted therapy may break immune tolerance, resulting in activation of immune cells and attack of cells expressing self-TAAs in normal tissues, causing autoimmune responses and autoimmune disease. TSAs are generated by nonsynonymous point mutations, deletion and/or insertions, gene fusions, and frameshift mutations that generate neoantigens. Neoantigens are only expressed in tumor cells, not normal tissue cells. They are ideal targets enabling T cells to recognize cancer cells. They can stimulate a strong antitumor immune response and are a major factor in clinical immunotherapy. Neoantigens can serve as biomarkers in cancer immunotherapy (1). The prerequisite for all treatments based on tumor neoantigens is the screening and identification of neoantigens (2). Therefore, identifying new antigens has become a top priority. The screening methods used to identify tumor neoantigens currently mainly include target gene sequencing (3), exome sequencing (4, 5), antigen ligandomics and mass spectrometry combination technology (4, 6, 7), and tandem micro gene sequencing methods (8, 9)(as shown in **Figure 1**).

**Figure 2:**
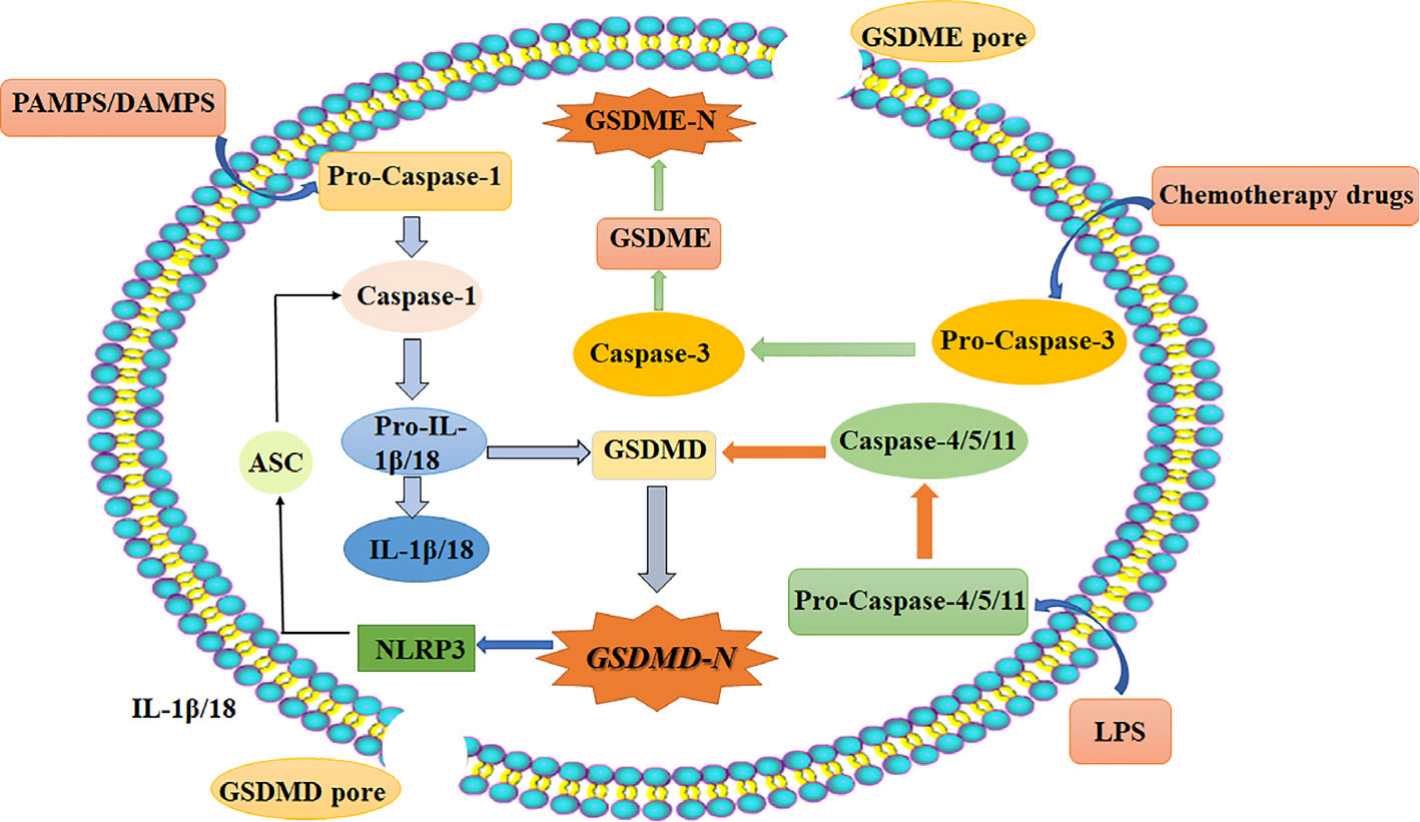
Schematic diagram of the three main pyroptosis pathways: Classical pathways that rely on caspase-1/NLRP3/GSDMD; Independent of caspase-1, through the nonclassical pathway caspase-4/5/11; (1). Bacteria, viruses, and inflammatory factors can activate NOD-like receptors (NLRs), absentin melanoma2 (AIM2) or pyrin domains (PYD) through pathogen-associated molecular patterns (PAMPs) or noninfectious stimulation-associated damage-associated patterns (DAMPs). (2). Lipopolysaccharide (LPS) could directly activate caspase-4/5/11, mediate the activation of GSDMD, activate NLRP3 inflammasome, and maturation and release of IL-1β and IL-18. (3). Chemotherapy drugs induce caspase-3 to cleave gasdermin E into GSDME-N and punch holes in the cell membrane to turn apoptosis into pyroptosis. Chemotherapy-induced caspase-3/GSDME specific pathways. DAMPs, damage-associated molecular patterns (noninfectious stimuli); PAMPs, pathogen-associated molecular patterns; LPS, lipopolysaccharide (gram-negative bacteria cell wall component); ASC, apoptosis-associated speck-like protein containing a CARD; IL, interleukin; NLRP3, NOD-like receptor protein 3; caspase, caspase family of proteins; GSDMD, gasdermin D.

**Figure 3:**
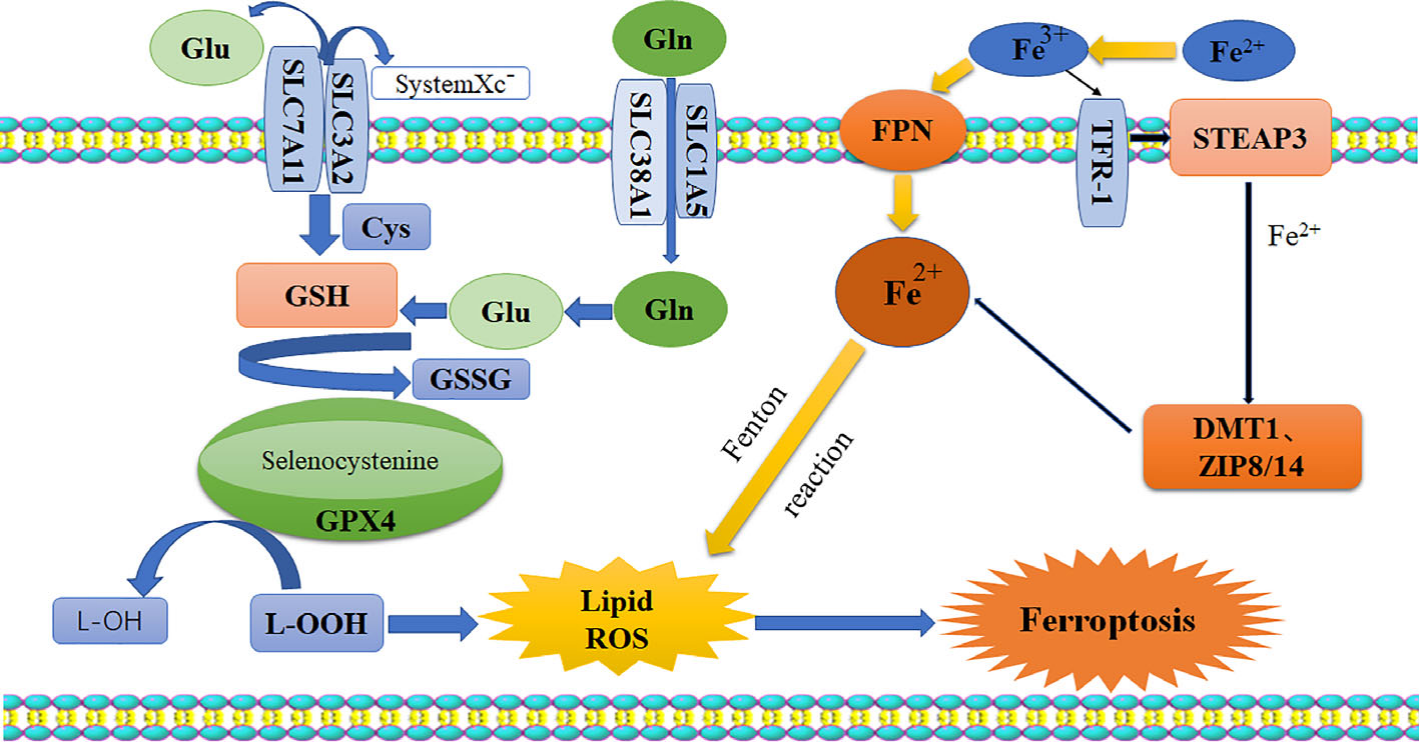
Schematic diagram of ferroptosis pathway. Glutamate and glutamine enter the cell body through system-xc, while Fe^2+^ enters the cytoplasm through FPN. Their accumulation would break the balance in the cells, and the lipid ROS is generated by GPx4 and Fenton reaction, resulting in ferroptosis. Glu, glutamate; Gln, glutamine; Cys, cysteine; GSH, reduced glutathione; GSSH, oxidized glutathione; GPX4, glutathione peroxidase 4; ROS, reactive oxygen species; FPN, ferroportin; TFR-1, transferrin receptor-1; STEAP3, six-transmembrane epithelial antigen of prostate 3; DMTI, divalent metal transporter; ZIP8/14, zinc transporter 8/14.

Pyroptosis is a kind of programmed cell death (PCD) characterized by proinflammatory and lytic properties, and it is also an important immune defense response of cells. Pyroptosis is mainly divided into the classical pathway, which depends on caspase-1 (cysteine aspartase-1) to mediate pyroptosis, the nonclassical pathway, which depends on caspase-4/5/11 to induce pyroptosis, and a pathway by which activation of tumor drugs induces caspase-3-mediated cleavage of GSDME into GSDME-N, which leads to pyroptosis. There are three pathways (10). It is now generally accepted that GSDMD is the executor of pyroptosis. GSDMD-N forms tiny pores of 1.0-2.4 μm in the cell membrane, which makes the ion gradient inside and outside the cell unbalanced. Water continuously pours into the cell until the cell ruptures, and inflammatory effectors flow out (IL-18/IL-1β), further enhancing the inflammatory response and ultimately leading to cell pyroptosis (11–13). The morphology of cells undergoing pyroptosis includes a cytoplasm that is preferentially located towards the surface, a centered nucleus, and a flattened shape that looks similar to cabbage or a fried egg (14). The specific mechanisms of pyroptosis are as follows (15, 16) (**Figure 2**): (I) Bacteria, viruses, and inflammatory factors can activate NOD-like receptors (NLRs), absentin melanoma2 (AIM2) or pyrin domains (PYD) through pathogen-associated molecular patterns (PAMPs) or noninfectious stimulation-associated damage-associated patterns (DAMPs). Such activity caspase-1, and then cleavage GSDMD to perform the occurrence of pyroptosis for GSDMD-N, and meanwhile activate nod-like receptor protein 3 (NLRP3). Apoptosis associated speck-like protein containing aCARD (ASC) further activates caspase-1. (II) Lipopolysaccharide (LPS) could directly activate caspase-4/5/11, mediate the activation of GSDMD, activate NLRP3 inflammasome, and maturation and release of IL-1β and IL-18. (III) Chemotherapy drugs induce caspase-3 to cleave gasdermin E into GSDME-N and punch holes in the cell membrane to turn apoptosis into pyroptosis. It is worth mentioning that caspase-1 promotes the maturation of inflammatory cytokines IL-1β and IL-18, and the GSDMD-N terminal releases inflammatory substances and induces pyroptosis. So these two proteins are crucial in pyroptosis. It has been shown that the expression of pro-death could be inhibited by the ROS scavenging agent N-acetylcysteine (17). And melatonin, which has a strong anti-inflammatory effect, could also prevent endothelial cell pyroptosis by down-regulating gene expression related to pyroptosis through the lncRNA-MEG3/miR-223/NLRP3 signaling pathway (18). Dolma (19) and others identified ferroptosis as a new form of PCD, and ferroptosis has become a research hot spot. Ferroptosis, mainly caused by the balance between the generation and degradation of reactive oxygen species in lipids, is an iron-dependent form of cell death caused by metabolic dysfunction of lipid oxides in cells under the action of divalent iron or ester oxygenase. High expression of unsaturated fatty acids on cell membranes and accumulation of lipid reactive oxygen (ROS) species result in an imbalance of intracellular redox. Studies have shown that inflammation is closely related to ferroptosis and has been shown to play an important role in the pathogenesis of aseptic inflammation (e.g., ischemia-reperfusion injury, stroke, and non-alcoholic hepatitis) as well as microbial infectious diseases (20–23). It is mostly in the following ways (24–26): 1. Glutamine (Gln) enters the cytoplasm through SLC38A1 and SLC1A5 of the cystine/glutamate antiporter system (system Xc-) and is then converted into glutamate (Glu). Free Glu also enters the cytoplasm through SLC7A11 and SLC3A2. Under the action of selenocysteine, Glu and cysteine work together to produce reduced glutathione (GSH). Then, oxidized glutathione (GSSG) is generated through the action of glutathione peroxidase 4 (GPX4). More importantly, under the action of glutathione peroxidase 4 (GPX4), L-OOH is deoxygenated to L-OH. This process leads to the accumulation of lipid ROS, which leads to ferroptosis. 2. The conversion of Fe^2+^ to Fe^3+^ and the direct conversion of free Fe^3+^ to Fe^2+^ can be accomplished under the action of ferroportin (FPN). Fe^3+^ can also enter cells through transferrin receptor 1 (TFR-1). Then, in the prostate, under the action of six-transmembrane epithelial antigen of prostate 3 (STEAP3), Fe^3+^ is reduced to Fe^2+^. Fe^2+^ is transported in cells by zinc transporter 8/14 (ZIP8/14) and divalent metal transporter 1 (DMT1). The resulting Fe^2+^ undergoes the Fenton reaction to induce the accumulation of lipid ROS, which leads to ferroptosis (**Figure 3**). Ferroptosis has unique characteristics in terms of cell morphology, genetics, and biochemistry. In terms of morphology, mitochondria become smaller in size, the membrane density increases, and cristae appear blurred or even reduced, and morphological changes in the nucleus are not obvious as major characteristics (27). In addition, in terms of biochemistry, the content of iron increases, ROS are produced in excess, and the expression levels of GSH and GPX4 are decreased. Furthermore, there are changes in some characteristics of genes (28). Studies show that iron chelating agents and antioxidants can inhibit the occurrence of ferroptosis, which can be induced by systemXc- inhibitors, GPXs inhibitors, and other compounds. The inductive mechanisms of ferroptosis in tumor cells can be divided into two parts. One is to induce ferroptosis by inhibiting the Xct/GSH/GPX-4 pathway axis, leading to the accumulation of lipid reactive oxygen species, and the other is to directly or indirectly induce ferroptosis around iron metabolism and mitochondria (27, 29).

With the advantage that neoantigens are specific to tumor cells, it is possible to cause tumor cells to undergo pyroptosis and ferroptosis or to change the tumor microenvironment and cause tumor cell death by targeting genetic mutations. Controlling and intervening in ferroptosis can delay or stifle the occurrence and development of tumors, improve the prognosis of patients with tumors and prolong survival, suggesting the value of relevant studies.

## Tumor Neoantigens And Pyroptosis

Pyroptosis plays an important role in many diseases, and it also has a certain role in the treatment of cancer. Recently, there have been few studies in related areas, but studies promoting or inhibiting the occurrence of pyroptosis to prevent the occurrence and development of tumors are warranted.

The emergence of high-frequency irreversible electroporation (H-FIRE) provides a new method for the treatment of tumors (30). H-FIRE can effectively ablate the primary tumor and induce the pro-inflammatory metastasis of the tumor microenvironment (31). The most important thing is, H-FIRE can produce tumor neoantigens; for example, H-FIRE produces 4T1 neoantigens in the treatment of breast cancer, which activates the adaptive immune system and significantly reduces tumor progression (31). H-FIRE induces acute inflammatory response through various pathways, an essential mechanism of antitumor immunity (32). Some studies show that in treating pancreatic cancer, H-FIRE combined with PD-1/PD-L1 shows that H-FIRE ablation is superior to thermal ablation and cryoablation in inducing T cell immunity, suggesting that H-FIRE ablation combined with immunotherapy may play a synergistic role. H-fire could directly punch holes in cells to induce K ^+^ outflow and induce pyroptosis (32). It is also found that even if a small number of tumor cells pyroptosis after treatment, they could chemotactic CD8^+^ T lymphocytes to accumulate in the tumor, thus effectively inhibiting tumor metastasis. Besides the stress state, the cells could induce pyroptosis through different signaling pathways (33, 34). Studies have found that *in vivo* and *in vitro*, the neoantigens produced by H-FIRE can interact with necrosis and pyroptosis-related cell death mechanisms through damage-related molecular signaling to kill tumor cells. Among these mechanisms, the pyroptosis of tumor cells may be due to the activation of NOD-like receptor (NLRS), absent in melanoma 2 (AIM2), etc., by noninfectious stimuli such as damage-associated molecular patterns (DAMPs). These events activate caspase-1, convert GSDMD to GSDMD-N, and promote the maturation and release of IL-1β and IL-18, causing pyroptosis to occur (35, 36). Studies show that STING (stimulator of interferon genes) agonists can activate NLRP3 by the caspase-1 pathway, thereby promoting the occurrence of pyroptosis and inhibit tumor progression in the Lewis mouse lung cancer model with neoantigen (37, 38). Therefore, it is speculated that STING agonists may inhibit the tumor by promoting pyroptosis of Lewis lung cancer cells with neoantigen.

In addition, a study found that retinoic acid can induce pyroptosis when activated by retinoic acid-inducible gene I (RIG-1) (39). RIG-1 is a receptor that recognizes abnormal viral mRNA in cells and is a member of the DexD/H box RNA helicase family (40). The C-terminus of RIG-1 contains the unwinding domain, which can interact with artificially synthesized double-stranded RNA and viral double-stranded RNA and unwind it in an ATPase-dependent manner. The N-terminus contains two sequential caspase activation and recruitment domains (CARDs). Once RIG-1 is activated, it can exchange its CARD for the CARD of the inflammatory molecule, causing the cell to rupture and activating the key molecule caspase-1, which in turn can cause maturation of the proinflammatory factors IL-1β and IL-18. The cleavage of the executor protein GSDMD, which also activates pyroptosis, into GSDMD-N leads to the occurrence of pyroptosis (41–43). Some studies have shown that in the cancer environment, RIG-1 signaling in tumor cells can affect the complexity of the TME (44); RIG-1 activation can provide many benefits for the treatment of tumors: 1, directly killing tumor cells; 2. activating innate immune effectors such as macrophages and natural killer cells through cytokines; and 3. enhancing the activity of professional antigen-presenting cells (APCs), such as dendritic cells (DCs) or macrophages by making the microenvironment rich in cytokines and increasing adaptive immunity by recruiting relevant cells (such as CD8+ T lymphocytes). Therefore, research on RIG-1 agonists is of extraordinary significance for the treatment of various cancers (45–48).

In summary, promoting pyroptosis could further activate the innate immune system, inhibit the development of tumor cells by changing the TME, and even directly kill tumor cells. Study shows that medical chemotherapy can activate the pyrodeath signal and induce pyroptosis. Furthermore, chemotherapy drugs can activate GSDME, a tumor candidate, to promote pyroptosis. This study provides a theoretical basis for chemotherapy combined with immune checkpoint therapy (16). Therefore, pyroptosis is a promising potential target for the treatment of cancer.

## Tumor Neoantigens and Ferroptosis

Ferroptosis is known to lead to the accumulation of ROS. In normal cells, the high accumulation of ROS is detrimental to the cell. However, tumor cells are a unique case. Studies have shown that intracellular ROS accumulation has both advantages and disadvantages for tumor progression (49). Compared with normal cells, tumor cells show significant upregulation of ROS. Therefore, to maintain REDOX homeostasis, tumor cells have evolved a powerful ROS scavenging system. This dynamic mechanism to ensure homeostasis enables ROS to act as promoters for tumor development and progression. Therefore, ROS induction therapy or antioxidant inhibitor therapy can modify ROS levels to induce tumor cell killing, which is a new strategy for the effective and selective killing of cancer cells (50).

Research shows that some tumor cells undergoing drug-induced death are very sensitive to ferroptosis, so ferroptosis has become a popular topic in tumor therapy research in recent years. For example, dihydroartemisinin can induce ferroptosis in head and neck squamous cell carcinoma cells (51), artemisinin (ART) can induce ferroptosis in pancreatic cancer cells by specifically inducing ROS production (52), and siramesine and lapatinib can induce ferroptosis in breast cancer cells (53). Therefore, inducing ferroptosis of tumor cells to inhibit tumor proliferation and metastasis may become a new strategy for tumor therapy in the future. A study showed that the cystine/glutamate antiporter SLC7A11 (commonly known as XCT), which is overexpressed in a variety of human cancers, could be used for GSH biosynthesis and antioxidant defense. By inhibiting cell ferroptosis, the overexpression of SLC7A11 promotes tumor growth. However, tumor cells with high SLC7A11 expression also undergo metabolic reprogramming associated with SLC7A11 overexpression, leading to the dependence of SLC7A11-overexpressing cancer cells on glucose and Gln, which provides a potential metabolic target for the therapeutic targeting of SLC7A11 in cancer (54). Erastin is a compound that has a strong inhibitory effect on cancers that express the RAS gene, and it can mediate ferroptosis (7). Therefore, developing strategies that can improve the content, solubility and potency of erastin is of great significance for cancer treatment (55–57). Nanoparticle-induced ferroptosis has also been demonstrated in xenotransplantation studies (58). It was confirmed as early as 15 years ago that the tumor suppressor gene p53 can inhibit SLC7A11 and, in some cases, can also induce ferroptosis (59). In addition, studies have confirmed that the nuclear transcription factor NRF2 can inhibit the occurrence of ferroptosis in tumor cells by regulating the transcription of SLC7A11, and inhibiting the expression of NRF2 can enhance cell ferroptosis (60). More interestingly, CD8+ cells that can recognize specific tumor antigens (neoantigens) and are involved in antitumor activities can secrete high levels of interferon-γ (IFN-γ), which is a cytokine that is important for CD8+ T cells to complete their immune killing function (61). Studies show that tumor neoantigens promote tumor cell killing by CD8+ cells (62). In addition, IFN-γ can downregulate the expression of two Glu-cystine antiporter subunits, recombinant solute carrier family 3, member 2 (SLC3A2) and SLC7A11, on the surface of tumor cells. Therefore, IFN-γ inhibits the uptake of cystine by tumor cells, reduces the synthesis of GSH in the cell, and ultimately leads to the insufficient synthesis of GPX4 in the cell, thus preventing the effective removal of lipid peroxides. Ion-dependent conditions lead to ferroptosis of cells (27, 63, 64). As mentioned above, tumor-specific therapy can stimulate CD8+ cells to release IFN-γ to induce ferroptosis of tumor cells without causing injury to normal cells.

In addition, molecules related to the NF2/YAP(neurofibromin 2/Yes-associated protein 1) signaling pathway, which are often malignant mutations in cancer, have also been found to play an important role in the regulation of ferroptosis. Therefore, malignant mutation events in molecules involved in the NF2/YAP signaling pathway could predict the sensitivity of cancer cells to iron-induced death therapy (65). Malignant mutations related to the NF2/YAP signaling pathway could also express related neoantigens on tumor cells. Therefore, in the future, it would apply the iron-chelating agents and antioxidants or SystemXC-inhibitors and GPXS inhibitors to regulate ferroptosis of tumor cells for malignant mutations related to the NF2/YAP signaling pathway. NF2/YAP signaling may be a potential target for the treatment of tumor cells.

## Application of Tumor Neoantigens

Advances in gene sequencing technology have enabled more tumor-specific immune targets to be discovered. In addition, tumor neoantigens that are only expressed in tumor cells can be recognized by the immune system, indicating that tumor neoantigens have a unique and important role in immunotherapy and warranting further development. Neoantigen-targeted tumor immunotherapy has already been actively attempted in the clinic.

### The CAR-T and TCR-T technology

Tumor neoantigen-based immunotherapy has enabled precise cellular immunotherapy for human tumors (66) and suggests the possibility of individualized treatment of cancer patients. T cells are lymphocytes that mainly mediate antitumor immunity, so strategies using T cells to specifically recognize tumor neoantigens to kill tumor cells are rational. Chimeric antigen receptor T cell (CAR-T) technology is an individual immunotherapy technology based on neoantigens. It is a tumor therapy strategy in which patients T cells are extracted and T cells with chimeric antigen receptors (CARs) with strong affinity for tumor cells are selected, ultimately enabling binding and killing of tumor cells. CAR-T cells are currently mainly used for hematological cancers. At present, CAR-T cells have been approved for the treatment of various CD19-positive hematological malignancies (67). The extraction of T cells from patients for CAR-T development is the component that makes CAR-T technology expensive. If allogeneic CAR-T cell treatment can be realized, it will have extraordinary significance for the treatment of solid tumors. Such treatment can overcome the challenge related to the release of excessive proinflammatory factors by T cells during tumor cell killing. Allogeneic CAR-T technologies are already in clinical trials, including CRISPR-based strategies to edit T cells. If the utility of allogeneic CAR-T treatment can be clinically verified, modified CAR-T can be produced on a large scale, which may reduce costs. However, studies have found that CAR-T therapy also exhibits “off-target” effects. In the treatment of colon cancer patients, the targeting of V-erb-b2 avian erythroblastic leukemia viral onco-gene homolog 2 (ErbB2) by CAR-T technology unexpectedly led to a large number of CAR-T cells in the lungs of patients, which resulted in cytokine release syndrome (CRS) and multiple organ failure (MOF) (68). The preparation of CAR-T targeting tumor-specific neoantigens may solve the problem of “off-target” effects. For now, clinical trials of CAR-T cells targeting ErbB2, disialoganglioside (GD2), and prostate-specific membrane antigen (PSMA) as new targets are also underway (69).

The T cell antigen receptor (TCR) is a marker of all T cells and can be used to identify histocompatibility complex (MHC) molecules and antigen peptide complexes carried by antigen-presenting cells (70). At present, the TCR is the only known molecule that can sensitively recognize epitopes on the surface of target cells. This unique ability also establishes the pivotal role of the TCR in T cell transfection technologies.

It has been found that modified TCRs have several advantages compared with CARs. CARs can recognize antigens in the molar range, and the target density required for the reaction needs to reach >10^3^ moles of antigen/cell. However, CARs can recognize peptides from the surface and intracellular proteins. They can also identify antigens in the micromolar range, and the target density required for the reaction only needs 1-50 moles of antigen/cell (71). Studies have shown that less than 25% of human proteins are membrane bound, and the proportion of amino acid sequences available on the cell surface (perhaps <10%) is low. Therefore, the number of antigens suitable for TCR targeting is much higher than that for CAR targeting (72, 73). CARs include a specificity determining region antibody fragment (single-chain antibody scFv variable region) that binds to specific antigens, a transmembrane domain (hinge/spacer domain, mostly from CD8) and multiple signal transduction elements (such as sequences from CD28, 4-1BB/OX40, CD3ζ and other important T cell molecules) (74–76); these domains determine the specificity of the CAR: surface proteins, glycoproteins, glycolipids, carbohydrates, or other antigens. However, the TCR recognizes the peptide-major histocompatibility complex (pMHC) complex inside and outside the cell. Therefore, these advantages are particularly important for the treatment of solid tumors. TCR targeting of tumor cells is an important strategy.

After the efficacy of CAR-T and TCR-T in treating hematologic malignancies, attention has shifted to CAR-engineered NK (CAR-NK) and TCR-engineered NK (NK-TCR) engineering. NK is a natural killer cell that has a strongly antitumor ability in the immune system. NK-TCR could improve the responsiveness and recognition specificity of NK cells to tumor cells (77). And CAR-NK has better safety and multiple mechanisms to activate cytotoxicity. NK cells also have a low risk of graft and host disease (GVHD) to be prepared in advance for use in multiple patients. CAR-NK cells can be designed to target multiple antigens, enhance proliferation and *in vivo* persistence, increase invasion of solid tumors, overcome drug-resistant tumor microenvironments, and ultimately achieve an effective antitumor response (78).

In summary, CAR-T and TCR-T technologies have similarities in the treatment of malignant tumors and are rapidly advancing, but ACT is the most promising treatment. The most important goal that must be accomplished for CAR-T or TCR-T technology to cure malignant tumors is identifying suitable target antigens. Although NK-CAR and NK-TCR technologies are not immature and have some technical and clinical challenges, their emergence offers more options and broad therapeutic prospects for cancer treatment.

### Anti-PD-1/PD-L1 Antibody Therapy

The programmed death receptor (PD-1) and its programmed death protein receptor-ligand (PD-L1) are called immune checkpoints, and they are important proteins for immune regulation. One study found that agents blocking cytotoxic T cell antigen 4 (CTLA-4) in combination with PD-1/PDL-1 blockade induced tumor regression in a portion of patients (79). This method is mainly aimed at activating the adaptive immune system in tumors with restricted checkpoint inhibition (80). Tumors with more mutations are likely to produce more new epitopes. These epitopes can be recognized by tumor-infiltrating T cells, but antibodies that block the checkpoint lead to tumor-infiltrating T cell activation and cause tumors to regress, so highly mutated tumors such as melanoma and lung cancer are more sensitive to anti-PD-1/PD-L1 antibody therapy (81, 82).

The first and most studied biomarker is PD-L1 protein expression. It is a potential biomarker of anti-PD-1/PD-L1 drug response. The clinical utility of immunohistochemistry (IHC) for detecting the expression of PD-L1 on tumor cells and/or tumor-infiltrating immune cells was confirmed initially in the first clinical study of the anti-PD-1 drug nivolumab and subsequently in other studies. The mechanisms of PD-L1 have been deeply studied (83).

The expression of PD-L1 is also closely related to prognosis. Some studies have collected information about PD-L1 overexpression in patients with gastric cancer, hepatocellular carcinoma, esophageal cancer, pancreatic cancer, ovarian cancer, and bladder cancer. PD-L1 expression has been shown to have an impact on prognosis in breast cancer and Merkel cell carcinoma patients, but the prognostic implications of PD-L1 expression are controversial for patients with lung cancer, colon cancer, and melanoma (84). For example, in kidney cancer, studies have found that the higher the expression of PD-L1 is, the more advanced the tumor stage and the worse the prognosis (85). Similarly, high expression of PD-L1 has a negative relationship with the survival rate of patients with non-small-cell lung cancer, but this idea has not been supported by clear evidence (86).

### Cancer Vaccines

The ideal tumor vaccine can efficiently induce humoral immunity and cellular immunity. While it is important to induce the proliferation and activation of specific T cells to enhance the killing of tumor cells *in vivo*, it is also necessary to prevent tumor vaccines from inducing recognition of TAAs that would trigger an autoimmune response. New antigen vaccines mainly include DC vaccines, peptide vaccines, DNA/RNA vaccines, antibody tumor vaccines, etc.

Because DCs are professional APCs, they can be used as effective inducers of tumor-specific immune responses (87). DC vaccines activate the immune functions of CD8 and CD4 T lymphocytes by loading tumor peptides and eliminate tumor cells. It was found that DCs can not only activate T cells but also maintain a balance between immune activation, suppression, and memory (88, 89). It is well known that DCs also recognize pathogen-related molecular patterns (PAMPs) and DAMPs through pattern recognition receptors (PRRs) (88), and pyroptosis is also induced by these molecular patterns. Strategies to inhibit the occurrence of DC pyroptosis, which could increase the number and quality of DCs to further kill and eliminate cancer cells, are worth considering.

**Figure 4:**
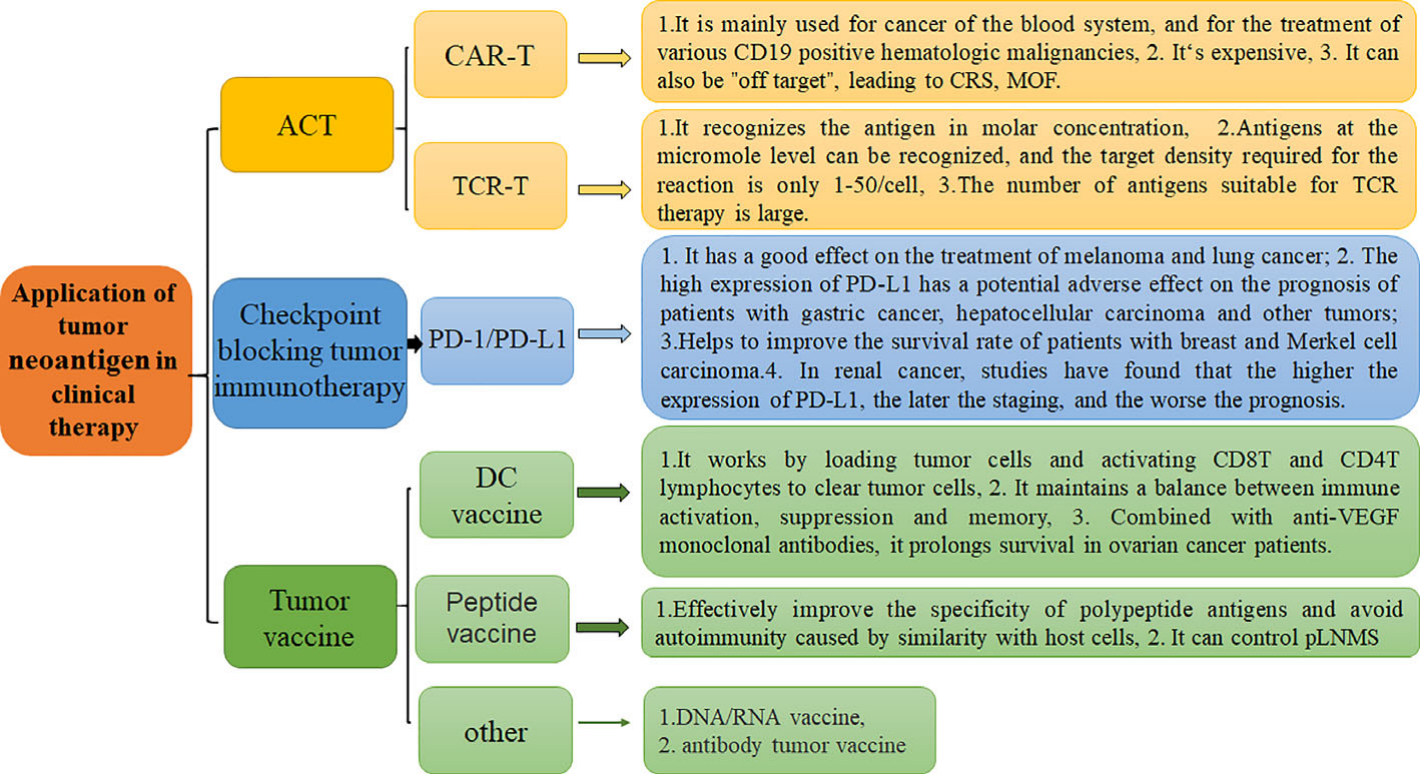
Application of tumor neoantigen-based strategies in clinical therapy and their respective characteristics. There are three primary clinical treatment methods, including ACT, Checkpoint blocking tumor immunotherapy, and tumor vaccine.

Antigen polypeptides eluted from the surface of tumor cells can be used to generate polypeptide vaccines, which direct immune cells to target abnormally expressed proteins inside tumor cells with strong specificity and safety. At the same time, further modification of amino acid residues or preparation of heat shock protein-peptide complexes can not only effectively improve the specificity of polypeptide antigens but also avoid autoimmunity directed at host cells. Studies have found that tumor peptide vaccines can control perihepatic tumor lymph node metastases (pLNMs), improve cancer prognosis, and increase the survival rate. The literature shows that combination of peptide vaccines with immune checkpoint inhibitor therapy may induce a synergistic effect to further improve the effects of peptide vaccines in clinical treatment (90) (**Figure 4**).

For example, tumor vaccine strategies have advanced, and tumor vaccines can be made according to individual differences. The development of such tumor vaccines has laid the foundation for individualized tumor treatment. DNA sequencing technology can be used to identify tumor-specific mutations. The sequence of a new antigen can be quickly inferred to make a vaccine. Once the cancer vaccine induces activation of specific T cells, anti-PD-1/PD-L1 antibodies can be used to enhance these immune responses against cancer cells (91). However, there are still many technical problems to be solved regarding tumor vaccines. For example, the vaccine preparation cycle is too long. In addition, it is necessary to sequence the genome of the patient’s tumor cells to identify relevant sequences to prepare the vaccine. Such a cycle may be too long for some advanced tumor patients. In addition, the cost makes this technology impractical.

## Discussion

As mentioned above, pyroptosis and ferroptosis have many effects on the occurrence, development, treatment, and survival of tumors. Pyroptosis and ferroptosis can kill tumor cells through different mechanisms. Therefore, these methods of PCD inhibit the occurrence and development of tumors and improve the survival rate of patients. Pyroptosis can also modify the anti-inflammatory tumor microenvironment *in situ* in a variety of ways to inhibit the growth and survival of tumor cells and can enhance the ability of APCs such as DCs and macrophages to activate the innate and adaptive immune systems. Pyroptosis can also enhance the recruitment of CD8+ T lymphocytes. The exchange of CARDs between RIG-1 and inflammatory molecules leads to the occurrence of tumor cell pyroptosis and delays the development of tumors. Research on the role of ferroptosis in the treatment of tumors has become more extensive. Studies have found that, on the one hand, tumor cell ferroptosis can be controlled, for example, by combined application of ROS inducers to regulate the level of ROS to treat pancreatic cell carcinoma (92). On the other hand, many drugs are able to induce ferroptosis of tumor cells.

Although the mechanism is not clear, they are likely to become new agents for future tumor treatments. However, the research on pyroptosis and ferroptosis in tumors is still not sufficient, and clinical applications are lacking, especially strategies including the combined targeting of pyroptosis, ferroptosis and tumor neoantigens for the treatment of cancer; such strategies are worthy of further investigation. For example, STING agonists could trigger pyroptosis, especially in Lewis mouse lung cancer cells with neoantigens. It may be due to the activation of caspase-1/NLRP3/GSDMD related pathway molecules by sting agonists. Simultaneously, the tumor cells of the Lewis mouse lung cancer model are more sensitive to sting agonists, leading to tumor pyroptosis. We can take advantage of this feature. It can make lung cancer cells pyroptosis and inhibit tumor progression.

Meanwhile, NF2/YAP is an essential molecular signal that regulates ferroptosis and is also a gene responsible for malignant mutations found in cancer. It is inferred that neoantigens produced by NF2/YAP mutated cancer cells can be identified by serological analysis techniques of recombinant expression cDNA clones, total exon sequencing (WES) combined with RNA sequencing (RNA-seq) and epitope prediction methods, and LC-MS/MS methods. To regulate NF2/YAP molecular signal to promote cancer cell ferroptosis, thereby inhibiting tumor occurrence and development and improving survival rate is possible. So using tumor cell neoantigens to target tumor cells and cause pyroptosis or ferroptosis is an important strategy for the future.

The accuracy of neoantigen screening methods needs to be improved, and the techniques need to be simplified to enable the development of neoantigen-based immunotherapies. Of course, reducing costs is also very important. Immunotherapy strategies related to neoantigens are rapidly being developed. The individualization of cancer treatment, prevention of treatment side effects, timeliness of treatment, and reduction of treatment costs are remaining challenges.

## Author Contributions

JY and QW, Writing-original draft. XC and ZG, Writing, review and editing. XZ and XC, Funding acquisition. All authors contributed to the article and approved the submitted version.

## Funding

This work was supported by the Natural Science Foundation of Shandong Province (grants ZR2020MH020, ZR2016CM20, and ZR2020MH415) and the National Natural Science Foundation of China (grants 81700406 and 81870237).

## Conflict of Interest

The authors declare that the research was conducted in the absence of any commercial or financial relationships that could be construed as a potential conflict of interest.

## Abbreviations

ACT, adoptive T cell therapy; AIM2, absent in melanoma; ART, artemisinin; ASC, apoptosis-associated speck-like protein containing Acard; CARD, Caspase activation and recruitment domain; Caspase, caspase family of proteins; CRT-T/TCR-T, chimeric antigen receptor T cell technology/T cell receptor engineering T cell technology; DAMPs, damage-associated molecular patterns (noninfectious stimuli); DMT1, divalent metal ion transporter; FPN, Membranotransferrin; GD2, disialoganglioside; GPX4, glutathione peroxidase 4; GSDME or E, Gasdermin E or D; GSH, glutathione; GSSG, oxidized glutathione; GVHD, graft and host disease; H-FIRE, High-frequency irreversible electroporation; IFN-γ, interferon-γ; IHC, immunohistochemistry; IIS, PD-1 or PDL-1 blockers; IL, interleukin; L-OH, the hydroxyl group of L-amino acids; LOOH, the carboxyl group of L-amino acids; LPS, lipopolysaccharide (gram-negative bacteria cell wall component); ASC, apoptosis-associated speck-like protein containing a CARD; MHC, histocompatibility complex; NLRP3, NOD-like receptor protein 3; NK-CAR, CAR-engineered NK; NK-TCR, TCR-engineered NK; PAMPs, pathogen-associated molecular patterns; PD-1/PD-L1, programmed death receptor/programmed death protein receptor ligand; pLNMS, pathological lymph node metastases; pMHC, peptide-major histocompatibility complex; PRR, pattern recognition receptor; PSMA, prostate-specific membrane antigen; PYD, pyrin domain; RIG-1, Retinoic acid-inducible gene I; ROS, reactive oxygen species; STING, stimulator of interferon genes; SystemXc-, cystine/glutamate reverse transporter system; TEM, tumor microenvironment; XCT, Cystine/glutamate antiporter; ZIP, Zinc transporter.
